# The relationship between parental health literacy levels and anthropometric measurements of children in Turkey

**DOI:** 10.1186/s12887-023-04385-4

**Published:** 2023-11-10

**Authors:** Ozcan Aygun, Mine Topcu

**Affiliations:** 1 Fethiye Health Science Faculty, Public Health Nursing Department, Mugla Sitki Kocman University, Calica Mevkii/Karaculha Fethiye, Mugla, Turkey; 2 Department of Nursing, Public Health Nursing Master’s Program, Institute of Health Sciences, Mugla, Turkey

**Keywords:** Anthropometric measurements, Child health, Health literacy, Health promotion, Parents health literacy

## Abstract

**Objective:**

The aim of this study was to identify the relationship between parental health literacy levels and anthropometric measurements of children in Turkey.

**Methods:**

The research was of cross-sectional/correlational design and carried out with 378 consenting parents registered at a Family Health Center. A Sociodemographic Data Form and the Health Literacy Scale for Turkey-32 was used to collect the study data. Data collection was completed at face-to-face interviews held in the consultation department of the family health center. The data were analyzed with the chi-square test and Ordinal Logistic Regression Analysis.

**Results:**

It was determined that parental health literacy levels were associated with level of education, income status and the state of the parents’ employment (p < .05). A relationship was also found between adequate levels of parental health literacy and the health status, weight and height standard deviation scores by age of the parents’ children (p < .05).

**Conclusion:**

This study found that adequate levels of parental health literacy were significantly lower than the European average. The study found that adequate parental health literacy had a positive effect on children’s anthropometric measurements. Health institutions and health professionals should plan training programs to improve the health literacy of parents when they apply to health institutions.

**Supplementary Information:**

The online version contains supplementary material available at 10.1186/s12887-023-04385-4.

## Background

The World Health Organization defines health literacy as the capacity to access information for the purpose of protecting and maintaining one’s health and the skills needed to understand, interpret and use the information gathered [1]. Health literacy in public health is one of society’s important indicators of the degree to which the individuals can benefit from health services, become aware of their right to health and public health services, understand health education materials, take medications carefully and accurately, and benefit from other aspects of healthcare [2]. It is ever more important today to ensure that individuals take responsibility for their own health, understand and interpret the information given to them, and make the right decisions about their health [3]. When health literacy is deficient in a society, the general level of health of the community suffers and problems arise in accessing and using health services, caring for and managing chronic conditions, maintaining the capacity or the will to practice self-care, among other issues [4].

Health literacy can have a positive or negative impact on parents in the context of their knowledge, attitudes and behavior with regard to accessing and benefiting from health services [5]. It is reported that parental health literacy levels have an impact on children’s growth and development and that the children of parents with inadequate health literacy are negatively affected. Inadequate health literacy has been shown to adversely affect a child in terms of eating habits, obesity, the correct administration of medications, application to emergency facilities and asthma management, oral and dental health, consumption of fruits and vegetables, physical activity and hygienic behavior [6–12]. Functional health literacy is said to be an important indicator of a child’s health and it is reported that breastfeeding self-efficacy levels rise with an increase in maternal health literacy levels [13, 14]. It is asserted that a mother’s level of education and the functional health literacy of parents are factors that impact children’s height, as well as their growth and development [15]. Conversely, inadequate health literacy has been associated with problems such as dwarfism, shortness and low weight [16].

It has been reported that among those in Turkey who prefer to seek medical services first at family health centers, 30.9% have an inadequate level of health literacy (HL), 38.0% have a problematic level of HL, 23.4% have an adequate level of HL, and 7.7% have an excellent level of HL [17]. In a study carried out in Turkey, it was found that the health literacy of parents who regularly carry out their own follow-up and follow-up of their children is higher than that of parents who have their children follow up when called by health care institutions [18]. In one study, the level of health literacy of parents had no association with their attitudes and behavior towards childhood vaccines [19]. Another study found that having a health problem for parents and their children increased their health literacy and that there was a relationship between parental health literacy and child health. To clarify this relationship, it is recommended to investigate the effect of parental health literacy on children’s health behavior [20]. It is stated that parental health literacy and body weight of adolescents are not related in Turkey [21], but no study was found that revealed the relationship between parental health literacy and anthropometric measurements of children under 6 years of age. International studies have shown that parents with a low level of health literacy are less likely to meet the needs of their children in terms of prevention and health care [7–9]. There is also evidence of an association between certain health behaviors in children that have a negative impact on their health, such as low parental health literacy, socioeconomic status, less healthy diet and less physical activity [11, 14–16].

Health care workers play an important role in helping communities’ access, understand and make use of health information. Public health and pediatric nurses should plan initiatives that will identify parental health literacy levels, which is a significant determinant of children’s health; they should seek to explore impacting factors, improve HL levels and ensure that parents gain the needed knowledge and develop the skills and attitudes conducive to maintaining pediatric health. In particular, nurses should guide parents and children to appropriate websites where they can find relevant health information, making sure that these resources provide accurate, reliable and updated information [22]. On the other hand, although there are many studies on parental health literacy and children’s health behaviors, there are few that inquire into the effect of parental health literacy on a child’s health behavior or the relationship between health literacy and children’s health [23]. We believe that this study will contribute to the literature by setting forth the relationship between levels of parental health literacy and children’s anthropometric measurements in Turkey.

The aim of this study was to identify the relationship between parental health literacy levels and anthropometric measurements of children in Turkey.

### Research questions

- What is the health literacy level of parents?
- What are the factors affecting the health literacy levels of parents?
- Is there a relationship between the health literacy levels of parents have an effect on children’s anthropometric measurements?

## Methods

### Sample and design

The universe of this cross-sectional study consisted of 538 parents with children of the ages 0–6 who were registered at a family health center. Using the G*Power program, sample size was calculated at a medium effect size, a 95% confidence level and a 5% margin of error, indicating that a total of 200 participants were needed for a power of 80% [24]. However, although our goal was to reach all the parents in the three-month data collection period, the study was completed with 378 parents with children of ages 0–6 who had been registered at the local family health center. The power of the sample to represent the universe was therefore 96%. In this study, parents who volunteered, had children aged 0–6 years, consented to their child’s height and weight being measured, and completed the data collection form in full were selected for inclusion in the study.

### Ethical considerations

Ethical approval was granted to the study by the Mugla Sitki Kocman University Health Sciences Ethics Committee (83/26.04.2021); institutional approval was obtained from the Aydın Provincial Health Directorate **(**327,897/15.09.2021**).** The staff at the family health center were informed about the study, and the consent of the parents regarding participation in the research was received. All of the stages of the study were conducted in keeping with the stipulations of the Helsinki Declaration.

### Measurements

A Sociodemographic Data Form and the Health Literacy Scale for Turkey-32 was used to collect the study data.

#### Sociodemographic data form

The sociodemographic data form prepared by the authors in line with the literature consisted of questions on the age, gender, civil status, level of education, condition of general health, employment status, economic status of the parents, and information about their child’s gender, age, body weight and height measurements [25]. The sociodemographic data form consists of 20 questions.

#### The health literacy scale for Turkey-32 (HLSTR-32)

The HLS-32 was derived from the 47-item European Health Literacy Scale [26, 27] and adapted to the Turkish language in the form of 32 items [28]. HLS-32 differs from the original scale in that it is based not on three, but two basic dimensions, on a 2 × 4 matrix. The scale has 4 options: 1 = Very easy, 2 = Easy, 3 = Hard, 4 = Very Hard. The statement “I don’t know” is coded as 5. Cronbach’s alpha coefficient for the scale was calculated as 0.927 [28]. In the evaluation of the scale, the indices have been standardized on a scale of 0–50, as in the HLS-EU instrument. The formula Index=(mean-1) x (50/3) was used for this. In this formula, the index is the index calculated unique to the individual and the mean refers to the mean of each item the individual responds to. The calculation provides a reading that indicates health literacy, 0 representing the lowest level of health literacy and 50 the highest. Scores of 0–25 signify an inadequate level of health literacy; >25–33, a level of problematic-limited, > 33–42, an adequate level; and > 42–50, excellent health literacy [26, 27]. The Cronbach alpha coefficient of the scale in this study was calculated as 0.978.

### Data collection

The data collection instruments were used through face-to-face interviews with the participants on weekdays in a room made available by the family health center for the research between the 1st of August and the 31st of October 2021. The researchers measured the weight and height of the infants and children using a weight scale and a height meter provided by the participants. This was done in a room provided by the family health center for the research. Once the measurement concluded, participants completed the data collection forms. The measurement of a child’s height and weight took about 5–8 min, and the completion of the data collection form by the parents took about 10 min. Then, the data were assessed by age according to the anthropometric measurements of Turkish children and standard deviation scores (SDS) were calculated and categorized for the children’s height, weight, and body mass index [29]. The SDS calculations according to age were assessed as follows [29]:

- *SDS for weight by age*: <-3 and − 2 underweight; > 3 overweight; 0, 1, 2 and <-1 normal.
- *SDS for height by age*: <-3 dwarf; <-2 short; >3 tall; 0, 1, 2 and <-1 normal.
- *SDS for body mass index by age*: >3 obese; >2 overweight; <-2 and − 3 underweight; 0, 1 and <-1 normal.

### Analytic strategy

The data obtained in the research were analyzed using the Statistical Package for the Social Sciences (SPSS 22) program. The data analysis made use of numbers, percentiles, and the Chi-square test. Ordinal Regression Analysis was used to assess the relationships between the sociodemographic characteristics of the parents and their health literacy levels and between the parents’ health literacy levels and their children’s anthropometric measurements. Statistical significance was accepted as p < .05 in all of the statistical analyses.

## Results

### Descriptive results

It was found that of the participants, 97.4% were women, 96.3% were married, 67.7% were gainfully employed, 69.3% were high school and university graduates, 61.4% had family income equal to their expenditure, 71.4% preferred to go to a family health center for an initial medical evaluation, 83.0% assessed themselves as in good or excellent health, and 94.2% said that the source of their knowledge about health was television. Of the parents, 85.2% had 3 children or fewer, the children of 87.8% did not have a chronic disease, and 82.6% assessed their child’s health to be good or excellent. The children’s standard deviation scores were accepted as normal for 77.2% in terms of weight by age, for 52.4% in terms of height by age, and for 86.2% in terms of body mass index by age. Of the parents, 34.9% displayed adequate health literacy (Table 1).

**Table 1:** Socio-demographic and individual characteristics of the participants

Variables	n	%
Gender of parents		
Male	10	2.6
Female	368	97.4
Age groups of parents		
19–29	162	42.9
Above 30	216	57.1
Martial status of parents		
Married	364	96.3
Single	14	3.7
Education level of parents		
Primary school	116	30.7
High school	140	37.0
University	122	32.3
Work status of parents		
Not working	256	67.7
Working	122	32.3
Income level of parents		
Income less than spending	34	9.0
Income equal to spending	232	61.4
Income more than spending	112	29.6
First healthcare provider of parents		
Primary health care	270	71.4
Hospital	108	28.6
Number of children of parents		
1	162	42.9
2	160	42.3
Above 3	56	14.8
Gender of child		
Male	214	56.6
Female	164	43.4
Parent’s level of assessment of health status		
Passing	64	16.9
Good	180	47.6
Very good	134	35.4
Parent’s level of assessment of the child’s health status		
Good	66	17.5
Very good	198	52.4
Excellent	114	30.2
Weight for age SDS level of child		
Underweight	68	18.0
Normal	292	77.2
Overweight	18	4.8
Height for age SDS level of child		
Stunted height	82	21.7
Short height	74	19.6
Normal height	198	52.4
Tall height	24	6.3
BMI for age SDS level of child		
Weak	20	5.3
Normal	326	86.2
Overweight/Obese	32	8.5
Health literacy level of parents		
Inadeguate	142	37.6
Problematic	104	27.5
Adeguate	132	34.9
Health information resources of parents		
TV	356	94.2
Internet	316	83.6
Social media	230	60.8
Friends	118	31.2
Health institutions	156	41.3
Scientific journal/book	22	5.8

### Comparison of various characteristics of the parental health literacy levels

It was observed that employed parents compared to unemployed parents and those that first applied to primary healthcare services compared to those that first applied to hospitals had higher and more significant levels of adequate health literacy (p < .001) (Table 2). A significant statistical difference was found between the parents’ level of health literacy and their level of education (x^2^ = 222.86 and p < .001), level of income (x^2^ = 113.23 and p < .001), the number of their children (x^2^ = 19.18 and p = .001), and their assessment of their own health (x^2^ = 69.80 and p < .001) (Table 2). The multiple comparisons between the parents’ levels of education and their health literacy levels showed that university graduates compared to both high school and elementary school graduates, high school graduates compared to elementary school graduates had higher and more significant levels of adequate health literacy (p < .001). In the comparison between the parents’ income status and their health literacy levels, it was noted that those with income exceeding their expenditure compared to those with equal income/expenditure and those with income less than their expenditure (p < .001), and those with equal income/expenditure compared to those with less income than expenditure (p = .020) had higher and more significant levels of adequate health literacy. It was seen that parents with one (p < .001) or two (p = .022) children compared to those with 3 or more children had higher and more significant levels of adequate health literacy. It was found that the parents who assessed their own health as excellent compared to those who assessed it as good or not bad, and those who assessed their health as good compared to those who assessed it as not bad had higher and more significant levels of adequate health literacy (p < .001).

**Table 2:** Comparison of sociodemographic and individual characteristics of the participants with general health literacy levels

Independent Variable	Health literacy levels			Chi-Square	p value
	Inadeguate %(n)	Problematic %(n)	Adeguate % (n)		
Gender of parents					
Male	20.0 (2)	40.0 (4)	40.0 (4)	1.50	0.472
Female	38.0 (140)	27.2 (100)	34.8 (128)		
Age groups of parents					
19–29	37.0 (60)	29.6 (48)	33.4 (54)	0.68	0.709
Above 30	38.0 (82)	25.9 (56)	36.1 (78)		
Martial status of parents					
Married	36.8 (134)	28.0 (102)	35.2 (128)	2.57	0.276
Single	57.1 (8)	14.3 (2)	28.6 (4)		
Education level of parents					
Primary school	77.6 (90)	20.7 (24)	1.7 (2)	222.86	**< 0.001**
High school	32.8 (46)	44.3 (62)	22.9 (32)		
University	4.9 (6)	14.8 (18)	80.3 (98)		
Work status of parents					
Not working	47.6 (122)	30.5 (78)	21.9 (56)	62.67	**< 0.001**
Working	16.4 (20)	21.3 (26)	62.3 (72)		
Income level of parents					
Income less than spending	76.5 (26)	5.9 (2)	17.6 (6)	113.23	**< 0.001**
Income equal to spending	44.0 (102)	36.2 (84)	19.8 (46)		
Income more than spending	12.5 (14)	16.1 (18)	71.4 (80)		
First healthcare provider of parents					
Primary health care	24.4 (66)	28.1 (76)	47.4 (128)	85.64	**< 0.001**
Hospital	70.4 (76)	25.9 (28)	3.7 (4)		
Number of children of parents					
1	27.2 (44)	28.4 (46)	44.4 (72)	19.18	**0.001**
2	42.4 (68)	26.3 (42)	31.3 (50)		
Above 3	53.6 (30)	28.6 (16)	17.8 (10)		
Parent’s level of assessment of health status					
Passing	68.8 (44)	18.7 (12)	12.5 (8)	69.80	**< 0.001**
Good	37.7 (68)	36.7 (66)	25.6 (46)		
Very good	22.4 (30)	19.4 (26)	58.2 (78)		

No significant relationship was detected between the parents’ levels of health literacy in terms of their age, gender, civil status, gainful employment status, the health facility to which they first applied, and the self-assessment of their health (Table 3). Being a university (OR = 43.905 and p < .001) or high school graduate (OR = 3.567 and p = .001) compared to being an elementary school graduate, and having an income that exceeded expenditure compared to an income that was less than expenditure (OR = 4.027 and p = .029), also having 1 child compared to having 3 or more children (OR = 1.346 and p = .007) were found to be factors highly and significantly associated with adequate levels of health literacy (Table 3).

**Table 3:** The assessment of sociodemographic and individual characteristics of the participants between general health literacy levels with Ordinal Logit Regression Analysis

Independent Variable	Estimate	S.E	Wald	OR	95% CI for OR		p value
					Lower	Upper	
Gender of parents							
Male	0.85	0.81	1.11	2.33	0.48	11.32	0.292
Female	0^a^						
Age groups of parents							
19–29	-0.05	0.41	0.01	0.95	0.42	2.13	0.910
Above 30	0^a^						
Marital status of parents							
Married	-0.75	0.65	1.32	0.47	0.13	1.70	0.251
Single	0^a^						
Education level of parents							
University	3.78	0.46	68.77	43.90	17.96	107.33	**< 0.001**
High school	1.27	0.37	11.59	3.56	1.71	7.41	**0.001**
Primary school	0^a^						
Work status of parents							
Not working	-0.16	0.30	0.28	0.85	0.473	1.53	0.594
Working	0^a^						
Income level of parents							
Income more than spending	1.39	0.64	4.74	4.02	1.15	14.11	**0.029**
Income equal to spending	0.11	0.53	0.04	1.11	0.39	3.15	0.836
Income less than spending	0^a^						
Number of children of parents							
1	1.08	0.40	7.32	2.94	1.35	6.41	**0.007**
2	0.57	0.39	2.17	1.77	0.82	3.80	0.140
Above 3	0^a^						
First healthcare provider of parents							
Hospital	-0.62	0.36	2.93	0.53	0.26	1.09	0.087
Primary health care provider	0^a^						
Level of self-assessment of parents							
Very good	-0.35	0.48	0.53	0.70	0.27	1.81	0.467
Passing	0.01	0.41	0.00	1.06	0.45	2.22	0.988
Good	0^a^						

S.E = Standart Error. OR = Odds ratio. Chi-Square = 270.04 df = 14 p < .001.

R-Square = Cox and Snell = 0.511. Link function: Logit.

^a.^ This parameter is set to zero because it is redundant

### The relationship between parental health literacy levels and children’s anthropometric measurements

A statistically significant difference was found between the parents’ health literacy levels in terms of their assessment of their children’s health (p < .001), the children’s weight SDS by age (p = .006), the children’s height SDS by age (p = .036) and the children’s body mass index SDS by age (p = .004) (Table 4). It was found in the multiple comparisons that parents assessing their child’s general health as excellent compared to parents who described this as very good (p < .001) or good (p < .001); as well as parents who described their child’s health as very good compared to those who described it as good (p = .011) had higher and more significant health literacy at the adequate level. It was discovered that parents with children whose weight SDS by age was normal compared to those whose weight SDS by age was underweight (p = .017), or those with children whose height SDS levels by age was short compared to those whose height SDS levels by age indicated dwarfism (p = .021), had higher and more significantly adequate health literacy levels. It was also found that parents with children with a normal mass body index SDS by age compared to children with a mass body index SDS by age indicating underweight (p = .009) displayed higher and more significantly adequate levels of health literacy.

**Table 4:** Comparison of parental health literacy levels and anthropometric measurements of children

Independent Variable	Health literacy levels			Chi-Square	p value
	Inadeguate %(n)	Problematic %(n)	Adeguate % (n)		
Parent’s level of assessment of the child’s health status					
Good	15.2 (10)	69.7 (46)	15.1 (10)	23.06	**< 0.001**
Very good	4.0 (8)	87.9 (174)	8.1 (16)		
Excellent	1.7 (2)	93.0 (106)	5.3 (6)		
Weight for age SDS level of child					
Underweight	52.9 (36)	20.6 (14)	26.5 (18)	14.41	**0.006**
Normal	32.9 (96)	28.7 (84)	38.4 (112)		
Overweight	50.0 (16)	31.3 (10)	18.8 (6)		
Height for age SDS level of child					
Stunted height	51.2 (42)	22.0 (18)	26.8 (22)	13.47	**0.036**
Short height	29.8 (22)	37.8 (28)	32.4 (24)		
Normal height	36.4 (72)	25.3 (50)	38.3 (76)		
Tall height	25.0 (6)	33.3 (8)	41.7 (10)		
BMI for age SDS level of child					
Weak	70.0 (14)	20.0 (4)	10.0 (2)	15.24	**0.004**
Normal	34.4 (112)	27.6 (90)	38.0 (124)		
Overweight/Obese	37.6 (16)	27.5 (10)	34.9 (6)		

BMI = Body Mass Index, SDS = Standart Deviation Score

Parents who assessed their children’s general health to be excellent (OR = 9.015 and pp < 0.001) or very good (OR = 2.817 and p = .001) compared to those that described it as good showed higher and more significantly adequate levels of health literacy. An association was found with higher and more significantly adequate levels of health literacy in the case of parents whose children displayed weight SDS indicating underweight (OR = 23.373 and p < .001) or normal weight (OR = 18.071 and p < .001) according to their age compared to parents with children whose weight SDS levels indicated overweight. An association was found with higher and more significantly adequate levels of health literacy in the case of parents whose children’s standard deviation scores indicated tallness for their age compared to parents with children with SDS indicating dwarfism by age (OR = 13.704 and p < .001) (Table 5).

**Table 5:** Evaluation of the relationship between parental health literacy levels and anthropometric measurements of children with Ordinal Logit Regression Analysis

Independent Variable	Estimate	S.E	Wald	OR	95% CI for OR		p value
					Lower	Upper	
Level of child self-assessment							
Excellent	2.20	0.36	37.53	9.01	4.46	18.21	**< 0.001**
Very good	1.04	0.32	10.30	2.81	1.49	5.30	**0.001**
Good	0^a^						
Weight for age SDS level of child							
Underweight	3.15	0.90	12.31	23.37	4.01	135.95	**< 0.001**
Normal	2.89	0.75	15.08	18.07	4.19	77.88	**< 0.001**
Overweight	0^a^						
Height for age SDS level of child							
Tall height	2.62	0.75	12.18	13.70	3.15	59.60	**< 0.001**
Normal height	0.38	0.43	0.75	1.45	0.62	3.407	0.385
Short height	0.55	0.40	1.87	1.73	0.79	3.80	0.171
Stunted height	0^a^						
BMI for age SDS level of child							
Weak	-0.52	0.70	0.54	0.59	0.15	2.35	0.461
Normal	0.01	0.46	0.00	1.01	0.41	2.49	0.978
Overweight/Obese	0^a^						

S.E = Standart Error. OR = Odds ratio. BMI = Body Mass Index, SDS = Standart Deviation Score, Chi-Square = 106.315 df = 15 p < .001. R-Square = Cox and Snell = 0.245. Link function: Logit.

^a.^ This parameter is set to zero because it is redundant

## Discussion

This study, which examined the relationship between parental health literacy and children’s anthropometric measurements, found that one third of parents had adequate health literacy and that these levels could be associated with education, income level and employment status. In addition, an association was found between the adequate level of health literacy of the parents and the Standard Deviation Scores (SDS) for weight and height of their children according to age. Several studies indicate that low levels of health literacy have an impact on parents’ knowledge, attitudes and behaviors, and also on children’s health outcomes such as disease prevention, acute care and chronic disease management. In addition, low health literacy has been shown to lead to negative outcomes such as poor nutrition knowledge and behavior, higher rates of obesity, more medication errors, and more visits to the emergency department [30, 31]. This study is the first in Turkey to show the effect of parental health literacy on children’s anthropometric measurements. It is also one of the few studies in the international literature. When reviewing the literature, it can be seen that parental literacy is mostly studied in areas such as children’s acute and chronic health problems, oral and dental health, mental health, emergency visits and caring for medically difficult children [6, 7, 10–12, 14, 23]. In this study, it is assumed that only 1/3 of parents have sufficient health literacy. This situation will have a negative impact not only on children’s anthropometric measurements, but also on acute and chronic health problems, mental health and positive health behaviors.

The research found that about two-thirds of parents had inadequate or problematic health literacy, whereas about one-third had adequate or exceptional health literacy. The level of adequate health literacy found in this study is similar to that found in some of the literature reviews [4, 32], but higher than that reported in other studies [33–35]. The prevalence of adequate health literacy in our study reflects the more favorable circumstances that exist in the study area compared to other regions of the country. The prevalence of adequate health literacy in our study reflects the more favorable circumstances that exist in the study area compared to other regions of the country. This is due to the higher level of education and income of the parents, as well as their level of health knowledge and use of preventive health services. The prevalence of adequate health literacy in our study reflects the more favorable circumstances present in the study area, in comparison to other regions in the country. Our study’s reported prevalence is higher than that of previous relevant research conducted in Turkey. However, it should be noted that the rate is comparatively lower than that reported in European countries. A study conducted in Europe found that only 52.7% of individuals had adequate and excellent health literacy [36]. This finding was supported by other studies that reported the rate of adequate and excellent health literacy in the population at 60-65% [37, 38]. These inadequate and problematic levels of health literacy highlighted in this study could potentially be attributed to factors such as economic instability, educational deficiencies, limited knowledge about health and other related gaps that exist in Turkey.

In this study, it was found that having parents with adequate health literacy was positively associated with their level of education, their level of income, their employment, their smaller family size and their self-assessment of their health. Some studies in the literature [39] report a significant correlation between health literacy and certain demographic factors, including age and level of education, with higher levels of health literacy found among women than men, those aged 65–74, married individuals, those with a high school education or higher, and those in employment [40]. Many studies in the Turkish literature [4, 33–35, 41] and international articles [37, 38] show consistency in the results regarding the level of health literacy among adults. The level of health literacy in a society is significantly influenced by factors related to employment, income and education [42–44]. Poor socio-economic conditions are recognized as contributing to low health literacy, and health literacy serves as a mediator in the relationship between the use of preventive health services and overall health status, quality of life and health behaviors [45]. The results of the study show that health literacy is significantly influenced by various socio-economic factors such as education, income and employment, as well as an individual’s ability to self-assess their health status.

We noted a correlation between possessing sufficient health literacy and parents’ ability to accurately evaluate their child’s health. Studies in the literature indicate that parents with high or functional health literacy exhibit preventative measures towards their children’s health [10, 46], possess greater understanding and expertise in managing their child’s asthma [47], instill their children with enhanced self-esteem [48], and adopt more constructive approaches towards their children’s health [13]. It is noteworthy for pediatric health that parents with sufficient health literacy were capable of reporting their child’s health as excellent or very good. Hence, a child’s well-being is influenced by the extent of their parents’ health literacy.

This study found no significant association between parental health literacy and a child’s body mass index. However, there is evidence to suggest that adequate parental health literacy may be associated with a child’s reduced likelihood of being unhealthily overweight and increased likelihood of being tall for their age. An important finding of this study is that rates of obesity and stunted growth were significantly lower in children of parents with adequate health literacy compared with those of parents with inadequate health literacy. A study has shown that low parental health literacy is linked to factors such as the socioeconomic status of the community, unhealthy dietary habits in children, and insufficient physical activity. These factors negatively affect a child’s health [9]. One study found an association between low parental health literacy and low parental self-efficacy in parents of new-born babies, which negatively affected their child’s health [49]. Another study found that parents with low health literacy put their children at increased risk of developing obesity, and that adequate parental health literacy had a positive effect on children, as they were more likely to adopt effective weight loss strategies and positive health behaviors [50]. There is evidence of a positive association between functional health literacy of parents and adult height of children, and adequate health literacy of parents has a positive impact on child development [15]. Some studies have shown a positive and statistically significant association between maternal nutritional literacy and children’s weight for age, height for age and body mass index [49, 51], and maternal nutritional literacy has been found to be effective in preventing stunting [52].

In this study, it is clear that there is a sufficiently positive relationship between having parents with adequate health literacy and assessing their children’s health, not being overweight for their age and being tall. It can be said that this situation may indicate that adequate parental health literacy may be effective in influencing positive health-related behaviors in children. Based on this situation, it can be said that adequate parental health literacy will positively influence anthropometric measurements of children aged 0–6.

### Strengths and limitations

This study is one of the few studies that has been conducted to determine the relationship between parental health literacy and children’s health. In this research explored the factors associated with parental health literacy as well as the relationship between parental health literacy and children’s health. It is important in terms of improving children’s health that health providers make an evaluation of the level of parents’ health knowledge and health literacy when they bring their children in for healthcare at primary care facilities.

The first and most important limitation to our study was that it was carried out in only one family health center. The second limitation is that all data on the data collection form other than the children’s measurements were based on self-reporting. Meanwhile, the fact that the study sample represented 70% of the study population and had a power of 96% were the strong points in the research.

## Conclusions

We found that the adequate health literacy of the parents we studied was higher than average for Turkey, but remarkably lower than the European average. In this study, it was found that parents’ adequate level of health literacy had a positive association with their level of education, employment status and income level. It also found that parents’ adequate health literacy was positively associated with their children’s health rating, weight for age and height for age, but not with the child’s body mass index for age. As a result, adequate parental health literacy has an impact on children’s anthropometric measurements. In light of these findings, we recommend that health care institutions and health professionals assess parents’ health knowledge and health literacy when they apply to health care institutions, and also implement practices that help to improve health literacy. In this context, educational programs to improve parents’ health literacy can be planned by health care institutions. Health care providers can reduce the impact of inadequate health literacy by trying to match families’ health care needs with their health literacy. It may also be beneficial to carry out more studies on the effects of parental health literacy on the health of children, and also on ways to increase the level of health literacy in general.

### Electronic supplementary material

Below is the link to the electronic supplementary material.

Supplementary Material 1

Supplementary Material 2

## Data Availability

The datasets used and/or analysed during the current study available from the corresponding author on reasonable request.

## References

[1] Nutbeam D, Levin-Zamir D, Rowlands G (2018). Health literacy and health promotion in context. Global Health Promotion.

[2] Nutbeam D, Lloyd JE. Understanding and responding to health literacy as a social determinant of health. Annual Review of Public Health. 2020; 42(2021): 159 – 73. 10.1146/annurev-publhealth-090419-102529. 10.1146/annurev-publhealth-090419-102529 33035427

[3] Van den Broucke S (2014). Health literacy: a critical concept for public health. Arch Public Health.

[4] Aygun O, Cerim S (2021). The relationship between general health behaviors and general health literacy levels in the Turkish population. Health Promot Int.

[5] Vamos CA, Thompson EL, Griner SB, Liggett LG, Daley EM (2019). Applying organizational health literacy to maternal and child health. Matern Child Health J.

[6] Abrams EM (2020). The impact of caregiver health literacy on pediatric Asthma: an integrative review. Pediatr Allergy Immunol Pulmonol.

[7] Brega AG, Johnson RL, Jiang L, Wilson AR, Schmiege SJ, Albino J (2021). Influence of parental health literacy on change over time in the oral health of American Indian children. Int J Environ Res Public Health.

[8] De Buhr E, Tannen A (2019). Parental health literacy and health behaviors in children: a 2017 cross-sectional survey in Germany. Eur J Pub Health.

[9] De Buhr E, Tannen A (2020). Parental health literacy and health knowledge, behaviours and outcomes in children: a cross-sectional survey. BMC Public Health.

[10] Lawrence PR, Feinberg I, Spratling R (2021). The relationship of parental health literacy to health outcomes of children with medical complexity. J Pediatr Nurs.

[11] Morrison AK, Glick A, Yin HS (2019). Health literacy: implications for child health. Pediatr Rev.

[12] Ricardo AC, Pereira LN, Betoko A, Goh V, Amarah A, Warady BA, Lash JP (2018). Parental health literacy and progression of chronic Kidney Disease in children. Pediatr Nephrol.

[13] Aydın D, Aba YA (2019). Annelerin sağlık okuryazarlığı düzeyleri ile emzirme öz-yeterlilik algıları arasındaki ilişki. Dokuz Eylül Üniversitesi Hemşirelik Fakültesi. Elektronik Dergisi.

[14] Lee HY, Zhou AQ, Lee RM, Dillon AL (2020). Parents’ functional health literacy is associated with children’s health outcomes: implications for health practice, policy, and research. Child Youth Serv Rev.

[15] Jarosz E, Gugushvili A (2020). Parental education, health literacy and children’s adult body height. J Biosoc Sci.

[16] Johri M, Subramanian SV, Kone GK, Dudeja S, Chandra D, Minoyan N, Pahwa S (2016). Maternal health literacy is associated with early childhood nutritional status in India. J Nutr.

[17] Ulusoy E, Yılmaz TE, Çifci A, Yılmaz T, Kasım İ (2020). Özkara,A. Sağlam çocuk takibinde ebeveynlerin rolü ve sağlık okuryazarlığı. Ankara Med J.

[18] Özkan S, Uğraş Dikmen A, Baran Aksakal F, Çalışkan D, Tüzün H, Taşçı Ö, Ceylan Ünal. S. Türkiye sağlık okuryazarlığı düzeyi ve ilişkili faktörleri araştırması. 2018; Retrived from https://sggm.saglik.gov.tr/TR, 2018, 56524.

[19] Ertuğrul B, Albayrak S (2021). Ebeveynlerin sağlık okuryazarlığı düzeyinin çocukluk dönemi aşılarına yönelik tutum ve davranışlarıyla ilişkisi. Hacettepe Üniversitesi Hemşirelik Fakültesi Dergisi.

[20] Alp S, Oral Kara N (2023). Ebeveyn sağlık okuryazarlığı ile pediatri hizmetleri kullanımını incelenmeye yönelik bir araştırma. Selçuk Üniversitesi Sosyal Bilimler Enstitüsü Dergisi.

[21] Ozturk Haney M (2020). Health Literacy and predictors of body weight in Turkish children. J Pediatr Nurs.

[22] Çınar S, Ay A, Boztepe H (2018). Çocuk sağlığı ve sağlık okuryazarlığı. Sağlıkta Performans ve Kalite Dergisi.

[23] Pawellek M, Kopf FM, Egger N, Dresch C, Matterne U, Brandstetter S. Pathways linking parental health literacy with health behaviours directed at the child: a scoping review. Health Promot Int. 2022;37(2). 10.1093/heapro/daab154. 10.1093/heapro/daab154 34668013

[24] Faul F, Erdfelder E, Lang AG, Buchner A (2007). G*Power 3: a flexible statistical power analysis program for the social, behavioral, and biomedical sciences. Behav Res Methods.

[25] Abacıgil F, Harlak H, Okyay P (2016). Türkiye sağlık okuryazarlığı ölçekleri güvenirlik ve geçerlilik Çalışması. Sağlık Bakanlığı Yayınları.

[26] Sørensen K, Van den Broucke S, Pelikan JM, Fullam J, Doyle G, Slonska Z, Brand H (2013). Measuring health literacy in populations: illuminating the design and development process of the European Health Literacy Survey Questionnaire (HLS-EU-Q). BMC Public Health.

[27] Sørensen K, Van den Broucke S, Fullam J, Doyle G, Pelikan J, Slonska Z, Brand H (2012). Health literacy and public health: a systematic review and integration of definitions and models. BMC Public Health.

[28] Okyay P, Abacigil F, Harlak H, Evci Kiraz ED, Karakaya K, Tuzun H, Beser E (2015). A new Health Literacy Scale: Turkish Health Literacy Scale and its psychometric properties: Pinar Okyay. Eur J Public Health.

[29] Neyzi O, Günöz H, Furman A, Bundak R, Gökçay G, Darendeliler F (2008). Türk çocuklarında vücut ağırlığı, boy uzunluğu, baş çevresi ve vücut kitle indeksi referans değerleri. Çocuk Sağlığı ve Hastalıkları. Dergisi.

[30] Costarelli V, Michou M, Panagiotakos DB, Lionis C (2022). Parental health literacy and nutrition literacy affect child feeding practices: a cross-sectional study. Nutr Health.

[31] Zaidman EA, Scott KM, Hahn D, Bennett P, Caldwell PH (2023). Impact of parental health literacy on the health outcomes of children with chronic Disease globally: a systematic review. J Paediatr Child Health.

[32] Guler DS, Sahin S, Ozdemir K, Unsal A, Uslu Yuvacı H (2021). Health literacy and knowledge of antenatal care among pregnant women. Health Soc Care Commun.

[33] Ayaz-Alkaya S, Ozturk FO (2021). Health Literacy Levels of Women and related factors in Turkey. J Nurs Res.

[34] Durmuş V (2021). Differences in health literacy level of patients from public and private hospitals: a cross-sectional study in Turkey. Public Health.

[35] Yiğitalp G, Bayram Değer V, Çifçi S (2021). Health literacy, health perception and related factors among different ethnic groups: a cross-sectional study in southeastern Turkey. BMC Public Health.

[36] Sørensen K, Pelikan JM, Röthlin F, Ganahl K, Slonska Z, Doyle G, Fullam J, Kondilis B, Agrafiotis D, Uiters E, Falcon M, Mensing M, Tchamov K, Van den Broucke S, Brand H, HLS-EU Consortium (2015). Health literacy in Europe: comparative results of the European health literacy survey (HLS-EU). Eur J Pub Health.

[37] Garcia-Codina O, Juvinyà-Canal D, Amil-Bujan P, Bertran-Noguer C, González-Mestre MA, Masachs-Fatjo E, Santaeugènia SJ, Magrinyà-Rull P, Saltó-Cerezuela E (2019). Determinants of health literacy in the general population: results of the Catalan health survey. BMC Public Health.

[38] Svendsen MT, Bak CK, Sørensen K, Pelikan J, Riddersholm SJ, Skals RK, Mortensen RN, Maindal HT, Bøggild H, Nielsen G, Torp-Pedersen C (2020). Associations of health literacy with socioeconomic position, health risk behavior, and health status: a large national population-based survey among Danish adults. BMC Public Health.

[39] Singh SG, Aiken J (2017). The effect of health literacy level on health outcomes in patients with Diabetes at a type V health center in western Jamaica. Int J Nurs Sci.

[40] Temel AB, Cimen Z (2017). Kronik hastalıgı olan yaslı bireylerde saglık okuryazarlıgı, saglık algısı ve iliskili faktorler. Ege Universitesi Hemsirelik Fakultesi Dergisi.

[41] Emiral GO, Tozun M, Atalay BI, Goktas S, Dagtekin G, Aygar H, Arslantas D, Unsal A, Babaoglu AB, Tirpan K (2021). Assessment of knowledge of metabolic syndrome and health literacy level among adults in Western Turkey. Niger J Clin Pract.

[42] Davis SN, Wischhusen JW, Sutton SK, Christy SM, Chavarria EA, Sutter ME, Roy S, Meade CD, Gwede CK (2020). Demographic and psychosocial factors associated with limited health literacy in a community-based sample of older Black americans. Patient Educ Couns.

[43] Goto E, Ishikawa H, Okuhara T, Ueno H, Okada H, Fujino Y, Kiuchi T (2020). Presenteeism among workers: health-related factors, work-related factors and health literacy. Occupational medicine (Oxford. England).

[44] Lastrucci V, Lorini C, Caini S, Bonaccorsi G, Florence Health Literacy Research Group (2019). Health literacy as a mediator of the relationship between socioeconomic status and health: a cross-sectional study in a population-based sample in Florence. PLoS ONE.

[45] Stormacq C, Van den Broucke S, Wosinski J (2019). Does health literacy mediate the relationship between socioeconomic status and health disparities? Integrative review. Health Promot Int.

[46] Aharon AA, Nehama H, Rishpon S, Baron-Epel O (2017). Parents with high levels of communicative and critical health literacy are less likely to vaccinate their children. Patient Educ Couns.

[47] Harrington KF, Zhang B, Magruder T, Bailey WC, Gerald LB (2015). The impact of parent’s health literacy on pediatric Asthma outcomes. Pediatr Allergy Immunol Pulmonol.

[48] Fong HF, Rothman EF, Garner A, Ghazarian SR, Morley DS, Singerman A, Bair-Merritt MH (2018). Association between health literacy and parental self-efficacy among parents of newborn children. J Pediatr.

[49] Cha E, Besse JL (2015). Low parent health literacy is associated with ‘obesogenic’ infant care behaviours. Evid Based Nurs.

[50] Liechty JM, Saltzman JA, Musaad SM, STRONG Kids Team (2015). Health literacy and parent attitudes about weight control for children. Appetite.

[51] Maheri M, Bidar M, Farrokh-Eslamlou H (2022). Evaluation of anthropometric indices and their relationship with maternal nutritional literacy and selected socio-economic and demographic variables among children under 5 years old. Ital J Pediatr.

[52] Sirajuddin SS, Razak A, Ansariadi, Thaha RM, Sudargo T. The Intervention of Maternal Nutrition Literacy has the potential to prevent Childhood Stunting: Randomized Control trials. J Public Health Res. 2021;10(2). 10.4081/jphr.2021.2235. 10.4081/jphr.2021.2235 PMC8129741 33855419

