# Supplementary material for: The relationship between parental health literacy levels and anthropometric measurements of children in Turkey
Source: BMC Pediatr. 2023 Nov 10;23:559. doi: 10.1186/s12887-023-04385-4 (PMC10636928; doi:10.1186/s12887-023-04385-4)
Supplement: Supplementary file 1 — Supplementary Material 1 [file 12887_2023_4385_MOESM1_ESM.pdf]

emine mizyal

Kuyu Sok. No. 7, Rumelihisarı
Istanbul, Turkey
Home Office: 90-212-263-6296
Mobile: 90-532-500-1724
E-mail: emizyal@gmail.com
Citizenship ID. 59587409478

TRANSLATION & INTERPRETATION
SERVICES

August 19, 2022

I provide this document to confirm and certify that I, Emine Mizyal Adsız, a native speaker of English and an independent translator, translated the manuscript with the English title

"The Relationship between Parental Health Literacy Levels and Children's Health Outcomes in a Family Health Center in Turkey"

from its original Turkish. Please note that I do not bear responsibility for revisions made to the document after my translation.

Emine Mizyal Adsız

Independent Translator,
Editor and Interpreter
