# Supplementary material for: The relationship between parental health literacy levels and anthropometric measurements of children in Turkey
Source: BMC Pediatr. 2023 Nov 10;23:559. doi: 10.1186/s12887-023-04385-4 (PMC10636928; doi:10.1186/s12887-023-04385-4)
Supplement: Supplementary file 2 — Supplementary Material 2 [file 12887_2023_4385_MOESM2_ESM.pdf]

MUĞLA SITKI KOÇMAN ÜNİVERSİTESİ
SAĞLIK BİLİMLERİ ETİK KURUL KARARI

Protokol No : 210042 **Karar No : 83**

**Araştırma Yürütücüsü**	Yüksek Lisans Öğrencisi MİNE TOPÇU
**Kurumu / Birimi**	MUĞLA SITKI KOÇMAN ÜNİVERSİTESİ / HALK SAĞLIĞI HEMŞİRELİĞİ
**Araştırmanın Başlığı**	Ebeveynlerin Sağlık Okuryazarlık Düzeyi İle Çocukların Büyüme Ve Gelişimleri Arasındaki İlişki
**Başvuru Formunun Etik Kurula Geldiği Tarih**	03.02.2021
**Başvuru Formunun Etik Kurulda İncelendiği Tarih**	İlk İnceleme Tarihi : **27.02.2021**<br>1. Düzeltme Tarihi : **14.03.2021**<br>2. Düzeltme Tarihi : **09.04.2021**
**Karar Tarihi**	**26.04.2021**

KARAR : **UYGUNDUR**
AÇIKLAMA :Beyan edilen veri formlarının dışına çıkılmaması şartıyla araştırmanın uygulanabilirliği konusunda bilimsel araştırmalar etiği açısından bir sakınca yoktur.

Prof.Dr. Haşim OLGUN
Başkan

Prof.Dr. Kılıçhan BAYAR
Üye

Prof.Dr. Nevin AKDOLUN BALKAYA
Üye

Prof.Dr. Özcan SAYGIN
Üye

Doç.Dr. Ahmet Salih SÖNMEZDAĞ
Üye

Doç.Dr. Cem ŞAHİN
Üye

Prof.Dr. Süleyman Cüneyt KARAKUŞ
Üye

Bu belge elektronik imza ile imzalanmıştır.
Doğrulama adresi https://etikkurulbasvuru.mu.edu.tr/dogrulama/UCFJ5316
